# Triple-Valve Nonbacterial Thrombotic Endocarditis Associated With Malignancy: A Rare Clinical Presentation

**DOI:** 10.7759/cureus.91031

**Published:** 2025-08-26

**Authors:** Thomas AD Jackson, Muhammad Khan, Anton Labeeb, Kashif Naeem, Susie Lewis

**Affiliations:** 1 Cardiology, Salisbury District Hospital, Salisbury, GBR

**Keywords:** endocarditis in malignancy, libman-sacks endocarditis, marantic endocarditis, multivalvular, non-bacterial endocarditis, sterile valvular vegetation, triple valve endocarditis

## Abstract

Nonbacterial thrombotic endocarditis (NBTE) is a rare form of endocarditis characterized by sterile vegetations, typically affecting the mitral or aortic valves. It is most commonly associated with hypercoagulable states, secondary to malignancy or systemic inflammatory disease. The condition itself is often asymptomatic and is frequently diagnosed when patients present with signs of systemic embolization. Heart failure is an uncommon initial presentation, and cases involving multiple valves are rare.

This report describes the case of a 57-year-old man who presented with progressive dyspnea and signs of cardiac failure. Imaging revealed a pulmonary embolism and a mass in the left lateral segment of the liver, suggestive of cholangiocarcinoma. The patient exhibited features of a hypercoagulable state, including two prior admissions for lower limb deep vein thromboses and signs of peripheral cutaneous ischemia. Transesophageal echocardiography (TEE) identified vegetations affecting the mitral, tricuspid, and aortic valves, each associated with severe regurgitation. In the context of malignancy and persistently negative blood cultures, a diagnosis of NBTE involving all three valves was made.

This case represents one of the rare instances of NBTE simultaneously involving the mitral, tricuspid, and aortic valves. While NBTE typically affects the left side of the heart, right-sided valve involvement is rare, and pulmonary embolism and heart failure are uncommon presenting features. This case underscores the importance of maintaining a high index of suspicion for NBTE in patients with malignancy who present with embolic events, new murmurs, or signs of heart failure.

## Introduction

Nonbacterial thrombotic endocarditis (NBTE) is a rare form of endocarditis, with a widely varied prevalence, from 0.3% to 9.3% in clinical reports. It is characterized by sterile vegetations, typically affecting the mitral or aortic valves [1]. It is most frequently associated with hypercoagulable states, particularly advanced malignancy, systemic autoimmune conditions [2], and, more recently, COVID-19 infection [3]. The most common types of malignancies linked with NBTE include pancreatic, lung (especially adenocarcinoma), gastrointestinal (such as gastric and colorectal), breast, and ovarian cancers, as well as hematologic malignancies like lymphoma and leukemia.

In malignancy, the mechanism by which these sterile fibrin-platelet vegetations accumulate is thought to involve a combination of elevated circulating cytokines, which cause endothelial damage on cardiac valves, and interactions between the tumor cells and macrophages, which promote clotting factor production and a hypercoagulable state [4].

Definitive diagnosis relies on the histological examination of valvular lesions to differentiate between sterile and infective endocarditis [1], but this is not a practical approach in patients unfit for surgery. Transesophageal echocardiography is a sensitive method for detecting vegetations [5], and when combined with persistently negative blood cultures, signs of systemic embolism, and an identified underlying cause, it can support a strong clinical diagnosis.

Treatment focuses on managing the underlying condition and initiating anticoagulation to reduce the risk of thromboembolism [6]. Some patients may be candidates for surgery, but in those with advanced malignancy, the risks often outweigh the potential benefits, and prognosis is typically limited by the primary disease [7].

## Case presentation

A 57-year-old man with a past medical history of coronary artery disease (percutaneous coronary intervention to the left anterior descending artery in 2013 and coronary artery bypass grafting in 2020), type 2 diabetes mellitus, and left ventricular dysfunction presented with progressive shortness of breath, orthopnea, and bilateral lower limb edema. He had two recent hospital admissions for lower limb deep vein thromboses in 2024 and early 2025.

On examination, the patient was tachypneic at rest with a dry cough, coarse crackles throughout both lung fields, and an ejection systolic murmur. Cardiovascular examination revealed elevated jugular venous pressure and pitting edema extending to the mid-thighs bilaterally. Peripheral examination identified a necrotic lesion on the left third toe and ischemic changes affecting the distal right third finger.

**Figure 1:**
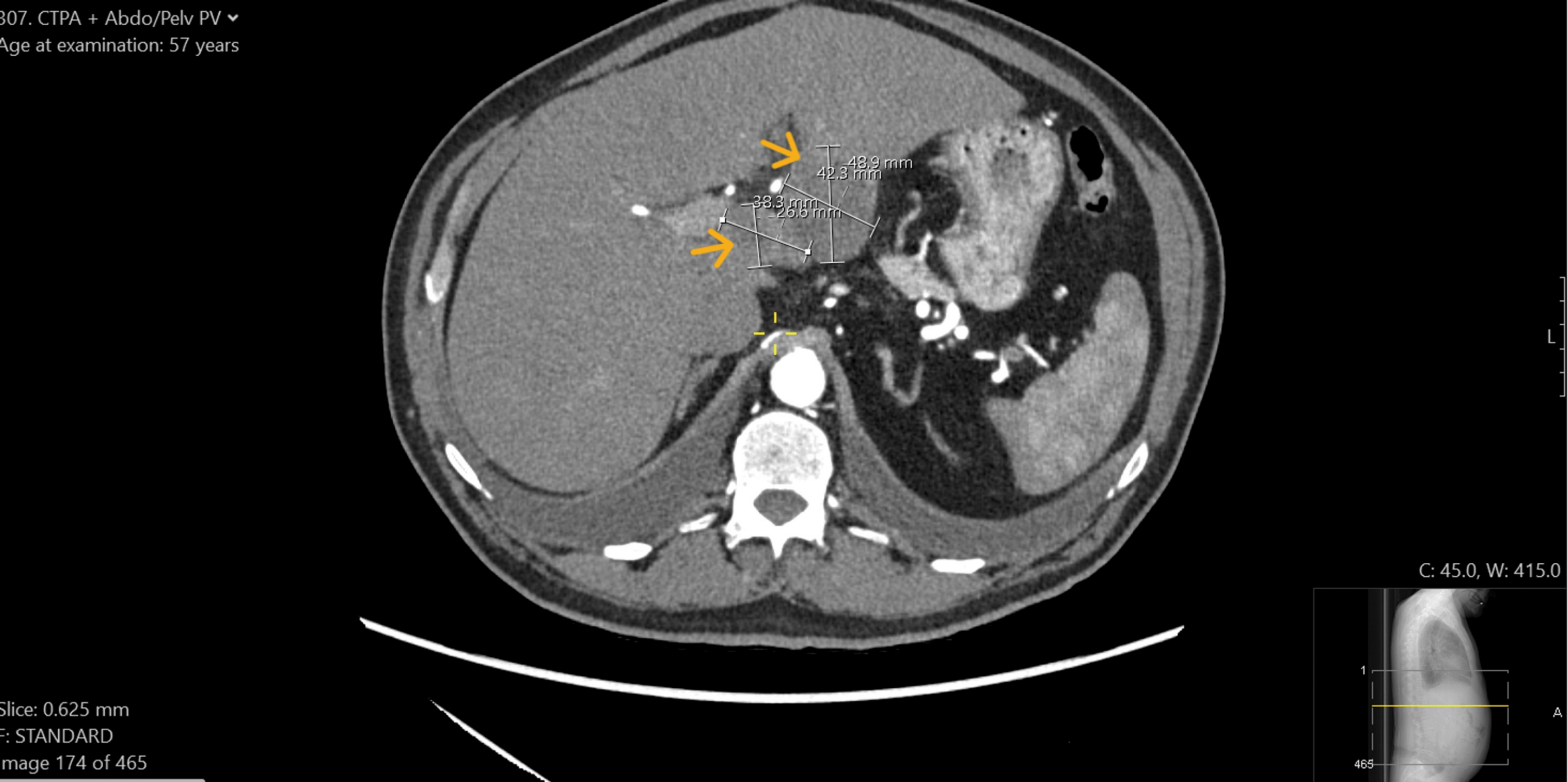
CT abdomen and pelvis Arrow indicating porta hepatis lymphadenopathy (left), and arrow indicating a mass in the left liver lobe (right).

**Figure 2:**
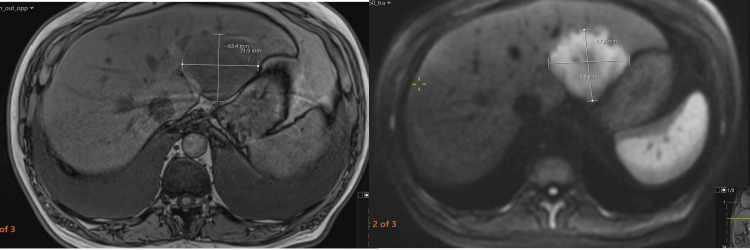
MRI T1 fat-saturated (left) and diffusion-weighted image (right) Arrows indicate a liver mass (left panel) and strong diffusion restriction on diffusion-weighted image (right panel).

Initial laboratory investigations demonstrated a normal white blood cell count, a mildly elevated C-reactive protein (CRP) level of 30 mg/L (reference range: 0.3-3.0 mg/L), which subsequently down-trended to less than 5 mg/L, and an elevated international normalized ratio (INR) of 6.2 (reference range: 0.8-1.2). An abdominal ultrasound revealed a mass in the left lobe of the liver, prompting further imaging. Computed tomography of the chest, abdomen, and pelvis (CTAP) identified pulmonary emboli in the right middle lobe of the liver. Treatment-dose of low molecular weight heparin was initiated after assessment by the hematology team, considering the significant thromboembolic events and the ongoing risk to the patient, despite an elevated INR. A CT scan of the abdomen and pelvis, along with magnetic resonance imaging (MRI) of the liver, confirmed a 7.1 × 6.3 cm mass in the left lateral segment of the liver (Figures 1, 2), diagnosed as cholangiocarcinoma. 

A biopsy was not pursued due to the patient’s underlying comorbidities, frailty, and the anticipated conservative management despite the presence of a definite histological diagnosis. The patient was referred to the gastrointestinal and surgical teams for further assessment.

Given his ongoing clinical signs of congestive heart failure, the cardiology team evaluated the patient. Transthoracic echocardiography demonstrated a severely reduced left ventricular ejection fraction and new severe mitral regurgitation, as indicated by echocardiographic parameters including a proximal iso-velocity surface area (PISA) of 0.96 cm (normal reference: <0.4 cm) and an effective regurgitant orifice area (EROA) of 0.41 cm² (normal reference: <0.20 cm²), along with suspicion of a mass or vegetation on the mitral valve. The study also revealed tricuspid valve vegetations and severe tricuspid regurgitation, evidenced by a visually severe eccentric jet, an elevated tricuspid regurgitation velocity (TR Vmax) of 4.1 m/s (reference: <2.8 m/s), and a markedly reduced right ventricular outflow tract acceleration time (RVOT Acc Time) of 60 ms (normal reference: 110-140 ms), indicating moderate to severe pulmonary hypertension.

**Figure 3:**
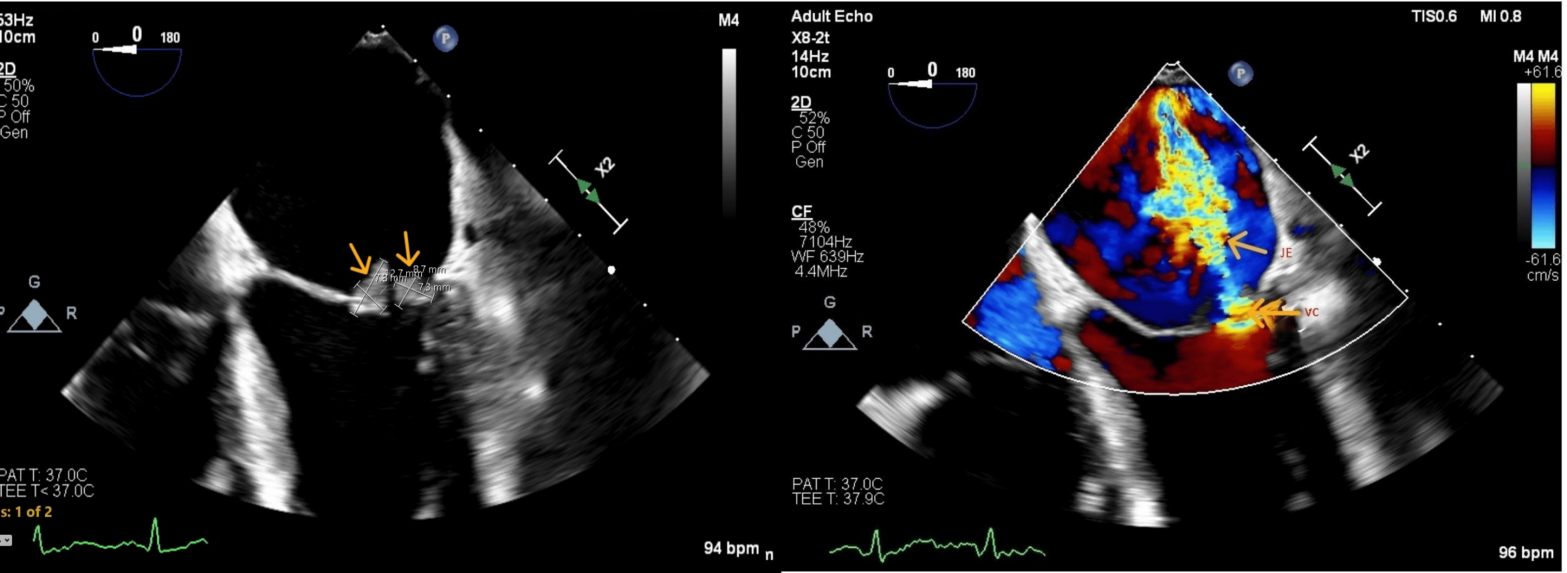
Transesophageal echocardiogram (mitral valve view) Arrows indicate mitral valve leaflet vegetations (left panel). Additional arrows highlight vena contracta (VC) and jet expansion (JE) on color flow Doppler, demonstrating mitral regurgitation (right panel).

**Figure 4:**
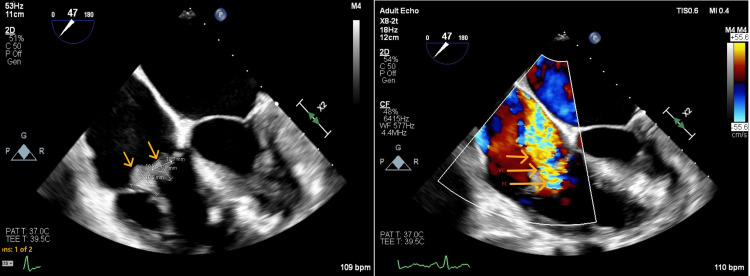
Transesophageal echocardiogram (tricuspid valve view) Arrows indicate tricuspid valve leaflet vegetations (left panel). Arrows also highlight flow convergence (FC), vena contracta (VC), and jet expansion (JE) on color flow Doppler, demonstrating tricuspid regurgitation (right panel).

**Figure 5:**
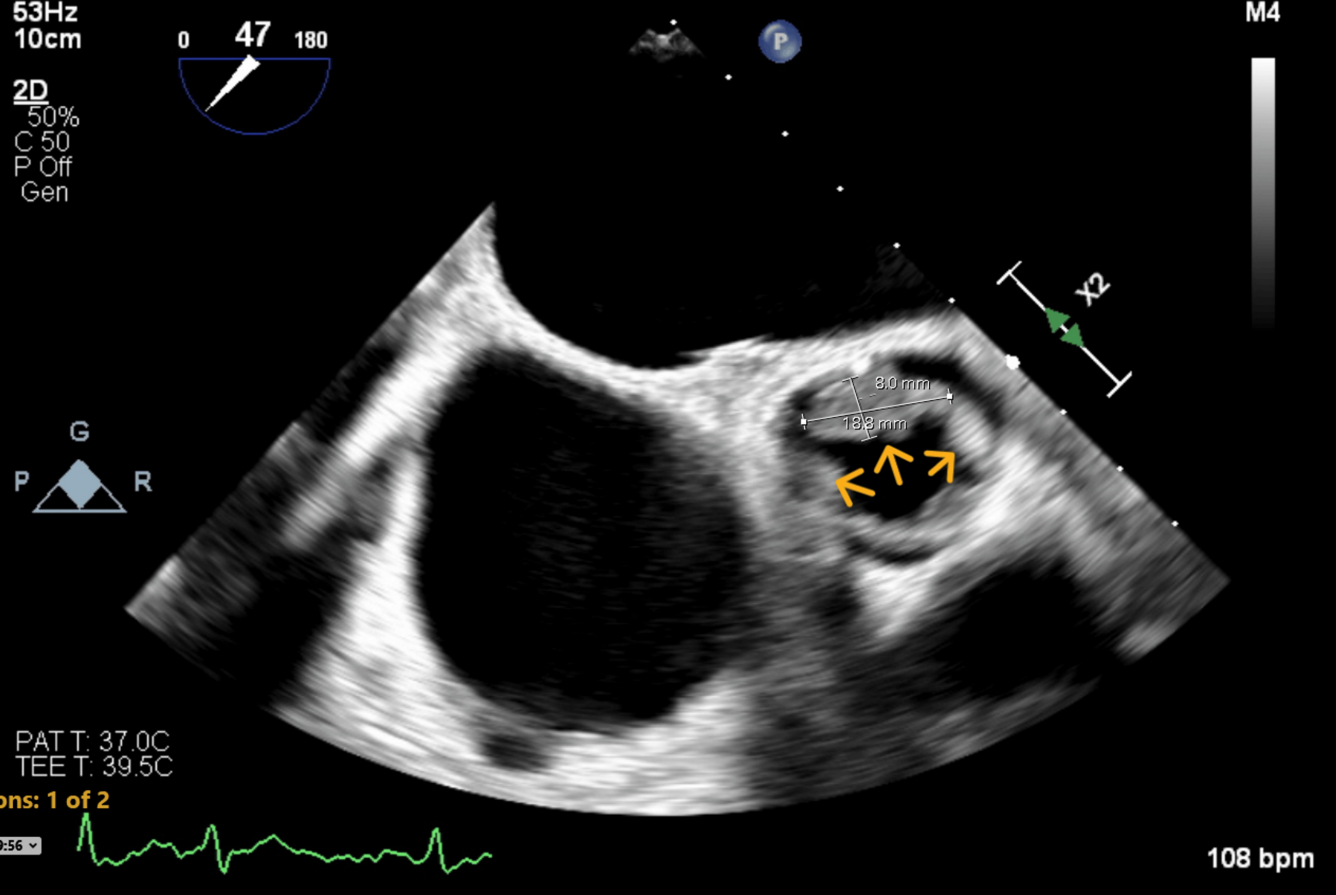
Transesophageal echocardiogram (aortic valve short axis view) Arrows indicate the aortic valve leaflet vegetation (center) and leaflet thickening (sides).

The aortic valve showed obvious thickening and vegetations on the leaflets, resulting in mild aortic stenosis and mild aortic regurgitation. A set of three blood cultures was obtained at this stage, and empiric antibiotics were initiated to cover possible infective endocarditis. A transesophageal echocardiogram (TEE) was subsequently performed, confirming vegetations on the mitral, tricuspid, and aortic valve leaflets, associated with severe mitral and tricuspid regurgitation (Figures 3-5).

Another set of blood cultures performed after TEE remained persistently negative.

To date, there is no single established set of diagnostic criteria for NBTE, in contrast to the Duke criteria [8] used for diagnosing infective endocarditis. Given the clinical context of advanced malignancy, persistently negative blood cultures, pulmonary embolism, and peripheral cutaneous ischemia suggestive of a hypercoagulable state, a diagnosis of NBTE involving three cardiac valves was made. After multidisciplinary team discussions, empiric antibiotics were stopped and the surgical intervention was deemed inappropriate due to the patient’s frailty, comorbidities, and advanced malignancy, all contributing to a poor prognosis. Management focused on symptom control with intravenous diuretics and anticoagulation, and the patient was transitioned to community-based palliative care.

## Discussion

NBTE most commonly affects a single left-sided valve, with the mitral valve being the most frequently involved [1]. It predominantly affects left-sided valves, owing to considerably higher shear stress, hemodynamic factors, and larger exposure to circulating prothrombotic stimuli. Right-sided involvement is relatively rare in NBTE unless specific risk factors (e.g., intravenous drug use or intracardiac prosthesis/devices) are present. Reports of multivalvular disease are rare, and to our knowledge, very few of the published cases have described simultaneous involvement of the mitral, tricuspid, and aortic valves. Our literature search, using broad inclusion criteria, identified several cases of dual-valve involvement [9-16], primarily mitral and aortic, while only three cases described involvement of both left- and right-sided valves, none of which were associated with malignancy [9-11]. The triple-valve involvement described in this case, therefore, represents a very rare anatomical distribution and is likely a marker of advanced systemic disease.

Vegetations in NBTE tend to be more friable than those in infective endocarditis due to lower levels of inflammation [4] and carry a higher risk of displacement and embolization; hence, anticoagulation is generally indicated in NBTE due to its prothrombotic nature. However, it is used cautiously or avoided in infective endocarditis due to a higher bleeding risk, especially if there are cerebral complications. In cases of NBTE, most patients present with embolic symptoms such as cognitive impairment secondary to cerebrovascular events, abdominal or flank pain due to mesenteric ischemia, renal artery thrombosis, or signs of deep vein thrombosis and peripheral ischemia [7]. In this case, the patient’s pulmonary emboli could be explained by the tricuspid valve vegetations, with further evidence of embolism including digital ischemia of the finger, a necrotic toe, and previous admissions within the last year for deep vein thrombosis.

The severe mitral, aortic, and tricuspid regurgitation observed is characteristic of the valvular insufficiency seen in the late stages of NBTE [4], explaining the patient’s clinical presentation of heart failure. There are no formal guidelines for surgical intervention in NBTE, and it is generally not recommended unless the patient is in acute cardiac failure [2]. In this case, however, given the poor prognosis of the underlying malignancy, a conservative management approach, comprising anticoagulation with heparin, diuretics, and palliative support in the community, was chosen.

Diagnosing NBTE in the context of malignancy remains a significant clinical challenge. The absence of pathognomonic features and overlap with infective endocarditis often delays recognition, particularly when systemic embolization is attributed solely to tumor-related hypercoagulability. TEE is an excellent modality for detecting vegetations, but it has limitations and cannot distinguish NBTE from infective endocarditis based on imaging alone. Diagnosis of NBTE is largely clinical, relying on TEE findings combined with negative blood cultures and underlying conditions such as malignancy or autoimmune disease [3]. In this case, TEE was critical in identifying vegetations that were not clearly evident on transthoracic imaging. While histological confirmation remains the gold standard, it is rarely pursued in patients with advanced cancer due to procedural risks and limited therapeutic consequences. This case highlights the need for heightened clinical suspicion of NBTE in patients with malignancy who present with embolic complications, culture-negative endocarditis, or new murmurs. Early recognition enables timely anticoagulation, supports shared decision-making about surgical intervention, and ensures appropriate palliative planning when curative treatment is not feasible.

## Conclusions

This case represents one of the rarely documented instances of nonbacterial thrombotic endocarditis involving the mitral, tricuspid, and aortic valves simultaneously. The co-occurrence of pulmonary embolism, peripheral thrombosis, and heart failure highlights the key diagnostic features and the severity of NBTE associated with advanced malignancy. Management of NBTE is focused on treating the underlying condition, anticoagulation to prevent embolic events and very rarely surgery if there is severe valvular dysfunction or recurrence. Prognosis is generally poor, as NBTE is often associated with advanced malignancy or systemic illness. Clinicians should maintain a high index of suspicion for NBTE in patients with cancer presenting with embolic events or new murmurs, particularly in the absence of positive blood cultures. As the documented prevalence of NBTE is relatively very low, there is considerable room for further research, registries, and refinement of diagnostic criteria to overcome major challenges posed by its rarity, under-diagnosis, and diagnostic ambiguity. 
